# Supplementary figures and images for: Regulation of stem/progenitor cell maintenance by BMP5 in prostate homeostasis and cancer initiation
Source: eLife. 2020 Sep 7;9:e54542. doi: 10.7554/eLife.54542 (PMC7525654; doi:10.7554/eLife.54542)

Full unedited gel for Figure 4S1B

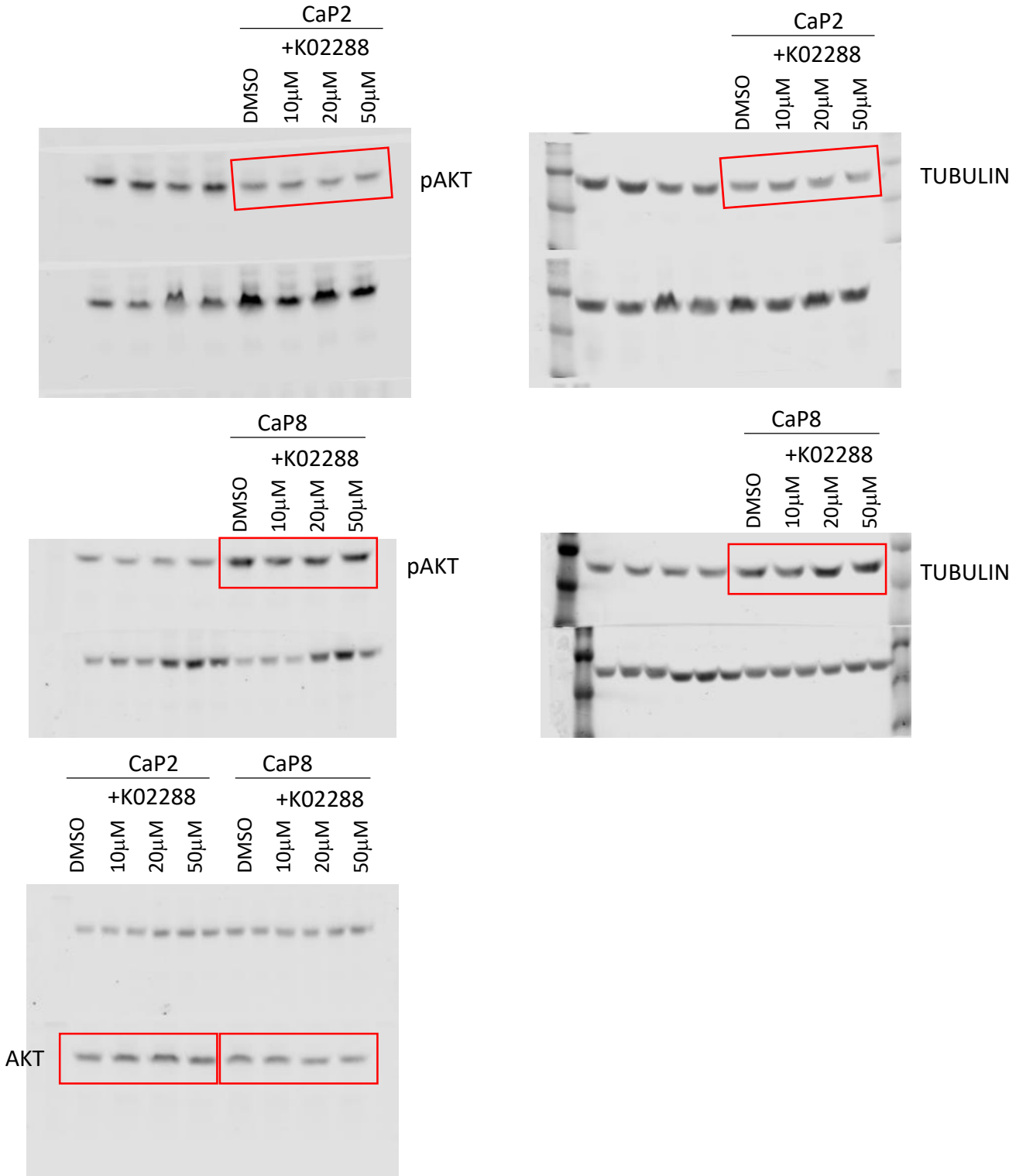

Supplement: Figure 4—figure supplement 2—source data 2. [file elife-54542-fig4-figsupp2-data2.pdf]
